# Manipulating the Mediator complex to induce naïve pluripotency

**DOI:** 10.1016/j.yexcr.2020.112215

**Published:** 2020-10-15

**Authors:** Cian J. Lynch, Raquel Bernad, Isabel Calvo, Manuel Serrano

**Affiliations:** a Institute for Research in Biomedicine (IRB Barcelona), The Barcelona Institute of Science and Technology (BIST), Barcelona, Spain; b Catalan Institution for Research and Advanced Studies (ICREA), Barcelona, 08010, Spain

**Keywords:** Pluripotency, Naïve, Primed, Mediator, Super-enhancers, Phase separation, CDK8, MEK, 2i, Demethylation, Embryonic stem, Reprogramming, RNA polymerase

## Abstract

Human naïve pluripotent stem cells (PSCs) represent an optimal homogenous starting point for molecular interventions and differentiation strategies. This is in contrast to the standard primed PSCs which fluctuate in identity and are transcriptionally heterogeneous. However, despite many efforts, the maintenance and expansion of human naïve PSCs remains a challenge. Here, we discuss our recent strategy for the stabilization of human PSC in the naïve state based on the use of a single chemical inhibitor of the related kinases CDK8 and CDK19. These kinases phosphorylate and negatively regulate the multiprotein Mediator complex, which is critical for enhancer-driven recruitment of RNA Pol II. The net effect of CDK8/19 inhibition is a global stimulation of enhancers, which in turn reinforces transcriptional programs including those related to cellular identity. In the case of pluripotent cells, the presence of CDK8/19i efficiently stabilizes the naïve state. Importantly, in contrast to previous chemical methods to induced the naïve state based on the inhibition of the FGF-MEK-ERK pathway, CDK8/19i-naïve human PSCs are chromosomally stable and retain developmental potential after long-term expansion. We suggest this could be related to the fact that CDK8/19 inhibition does not induce DNA demethylation. These principles may apply to other fate decisions.

## Graphical abstract

**Figure:**
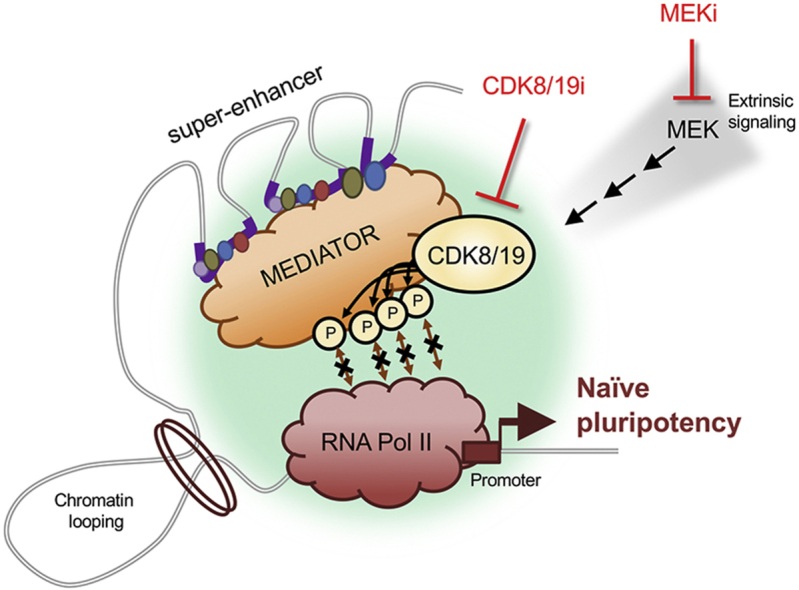

## 1 A new approach to human pluripotency

Human embryonic pluripotent stem cells (PSCs) provide a challenging parallel to our understanding of pluripotency based in rodents. The extent to which human PSCs can be captured in a stable naïve state, its similarity to rodent naïve pluripotency, and even its clinical usefulness, remain in question [[1], [2], [3]]. Inhibition of the FGF-MEK-ERK signaling pathway is central to naïve pluripotency across multiple mammalian species, however the application of this in culture media appears to require species-specific adaptations to avoid genomic instability [[4], [5], [6], [7]]. Understanding the mechanisms by which MEK-inhibition leads to the transition from primed to naïve pluripotency remains in progress.

**Fig. 1:**
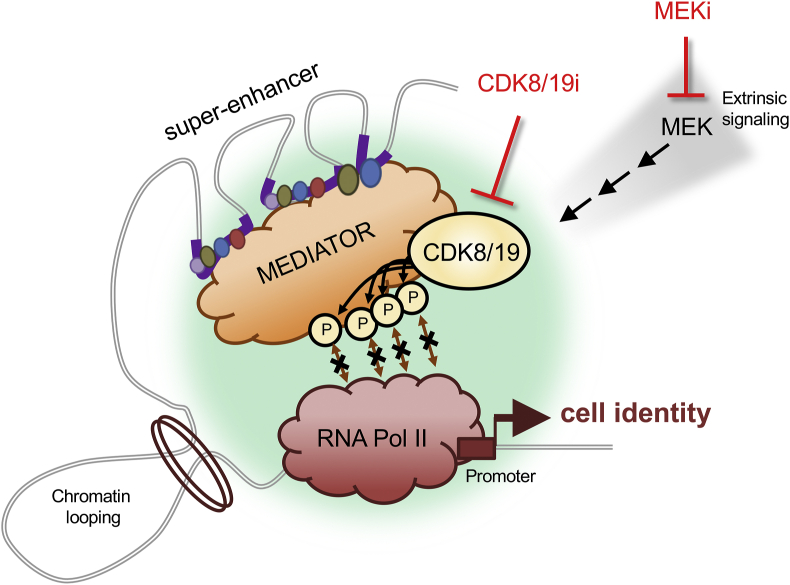
**The Mediator complex integrates multiple signals and bridges enahcers to promoters to regulate pluripotent cell identity.** Mediator acts as a central hub which links enhancers and target promoters, integrates combinatorial signals, and recruits RNA Pol II, to guide the transcriptional program of cell identity. The core transcriptional machinery is shown simplified, within a phase-separated transcriptional condensate (light green, membrane-less region). The largest enhancers, known as super-enhancers emerge by coalescing multiple smaller constituent enhancer regions (purple thick lines), each enriched in lineage-specifying transcription factors, histone marks, and chromatin regulators (indicated by small coloured circles). Mediator associates with these many factors and receives upstream signaling inputs from pathways, including MEK-ERK. Mediator recruits RNA Pol II to the pre-initiation complex, a function which can be hindered by the CDK8/19 kinase-module, via structural or kinase-dependent activities of CDK8/19. In PSCs, the 2i inhibitor cocktail is known to rapidly alter the transcriptional program to promote naïve pluripotency, of which, MEK-inhibition is the key feature. However, how MEK-inhibition resets the transcriptional machinery to the naïve program remains to be clarified. In PSCs, we find that MEK-inhibition leads to global up-regulation of enhancer activity via increased RNA Pol II recruitment. We find that the reorganization of the transcriptional machinery by MEK inhibition operates largely via controling the activity of CDK8/19 downstream. In this way, both treatments induce a highly overlapping set of phospho-changes focused on the transcriptional machinery, hyper-activating enhancers and Mediator, triggering increased RNA Pol II recruitment, and promoting the transcriptional program of naïve pluripotency.

The Mediator complex operates at enhancers and is a pivotal orchestrator of the transcriptional program of cell identity [8,9] (Fig. 1). Moreover, several data suggest that Mediator function may lie downstream of the MEK-ERK pathway (see below). Indeed, we recently identified an important role for the Mediator complex in stabilizing naïve pluripotency both in mouse and human PSCs, which recapitulates many aspects of MEK-inhibition [10]. In this review, we introduce the role of Mediator in cell identity, in particular during pluripotency, and we summarize our recent report of a pharmacological strategy for the manipulation of Mediator that stabilizes the naïve state in mouse, human, and non-human primate PSCs [10]. We also discuss the implications for other states of cellular plasticity beyond pluripotency, and its application in directing the resolution of cellular fate decisions.

## 2 Enhancers, super-enhancers, and cell identity

Each cell-type contains a unique repertoire of active enhancer complexes at specific DNA regions, which arise by high concentration of lineage-specific transcription factors [[11], [12], [13], [14]]. Collectively, the transcription factors, associated chromatin regulators, and the epigentic marks that they generate, serve as the platform to recruit a single large multi-protein complex known as Mediator [8,9,15] (Fig. 1). In a sense, the diverse combinatorial information input present at enhancers is reduced into a single output, namely, the Mediator complex, which thereby can be considered the universal transducer of enhancers. The main function of Mediator is, in turn, the recruitment of RNA polymerase II (RNA Pol II) to nearby and distant promoters, thus having a major contribution to the transcriptional program characteristic of each cell type [8,9,13,16] (Fig. 1).

Super-enhancers (SEs) constitute a relatively novel concept that refers to a small fraction of unusually large and powerful enhancers [12,13]. SE's are multipartite, as they arise by coallescing clusters of smaller typical enhancer constituents in close proximity, and these large aggregates display emergent properties that distinguish them functionally from the smaller typical enhancers (TEs) [17] (Fig. 1). In particular, SEs are extraordinarily potent in driving transcription and they rely on phase separation to confer their stability and flexibility to change [17]. Interestingly, the most influential genes determining cell identity are often found under the control of SEs [[11], [12], [13]]. SEs drive high expression of master transcription factors, which in turn, enrich within the enhancer loci, completing a positive-feedback loop and establishing the regulatory network that maintains cell identity [[11], [12], [13]]. In agreement with its critical role in recruiting RNA Pol II, Mediator is highly abundant at SEs [13] and therefore, we reasoned that it could be an actionable point to manipulate SEs and cell identity (Fig. 1). Below, we summarize the current understanding of Mediator function, and regulation by CDK8 and CDK19 kinases (abbreviated as CDK8/19).

## 3 Mediator: a bridge between enhancers and promoters

There have been great advances in recent years regarding the structure and mechanistic functioning of Mediator [8,9,15,[18], [19], [20]]. The 30 subunits of Mediator are organized in four general domains: the “head”, “middle”, and “tail” domains that constitute “core-Mediator”, plus a fourth accessory domain known as the “CDK8/19-module” (Fig. 1). The Mediator tail-domain interacts with the enhancer chromatin, including transcription factors and cofactors, while the middle- and head-domains form contacts with RNA Pol II and the pre-initiation complex at target promoters [8,18,19]. While the many subunits of Mediator can undergo extensive structural re-arrangements, the CDK8/19-module contains the only enzymatic activity of Mediator, namely the kinase CDK8 or its highly similar, but poorly studied, paralog CDK19 [9,21,22]. Completing the kinase module are: cyclin C (CCNC), and subunits MED12 and MED13. CDK8/19 activity appears restricted by its requirement for proper quaternary structure [23]. For full activity, CDK8/19 must associate simultaneously with cyclin C and with MED12, the latter embraces CDK8 allowing it to attain proper opening of its T-loop [23]. Evidence suggests that only CDK8, or CDK19, can occupy Mediator at one time, and the same is true for the paralog subunits MED12L and MED13L (reviewed elsewhere) [8,9,22]. Collectively, these may represent alternate combinations of the CDK8/19-module, providing opportunity for subtle modulation of Mediator function.

The Mediator complex must associate with the many different transcription factors that define an enhancer locus. This constitutes one arm of the Mediator bridge at the enhancer. Recent evidence hints at how this may be achieved. Mediator subunits in its tail sub-module have been shown to engage in a high number of low specificity and weak interactions via phase separation, thereby achieving a “fuzzy” interaction between Mediator and the activation domains of many different transcription factors [[24], [25], [26], [27]]. The second arm of the Mediator bridge extends to interact with the pre-initiation complex, inducing the recruitment of RNA Pol II at target promoters [20] (Fig. 1). Comprehensive cryo-EM studies and *in vitro* biochemical assays have collectively revealed how Mediator undergoes extensive structural re-arrangements to achieve this [8,9,18,19]. Notably, the CDK8/19-kinase module plays a key role, associating with core-Mediator and repressing RNA Pol II recruitment [8,9,18,19] (see below). In addition to its structural aspects, the Mediator bridge is dynamic and programmable, integrating many upstream signals [8,28]. Precisely how upstream signals modulate Mediator function is only recently emerging. Acetylation [29] and phosphorylation [10,[30], [31], [32], [33]] of multiple sites and subunits of Mediator have been detected, implicating p300 acetyl-transferase activity, and the MEK-ERK pathway, as major inputs.

## 4 CDK8/19: the sub-module of Mediator that represses RNA Pol II recruitment

The CDK8/19-kinase module (CDK8/19-CCNC-MED12/L-MED13/L) associates with core-Mediator and plays a central role in the regulation of how Mediator recruits RNA Pol II during each transcription cycle [8,9,22]. The detailed mechanisms of how CDK8/19 operates during the transcription cycle remain to be fully understood [22,34], and the relative contribution of CDK8/19 kinase activity versus its kinase-independent structural effects are unclear. However, we and others have observed that the ultimate outcome of CDK8/19 kinase-inhibition is increased recruitment of RNA Pol II by Mediator, and stimulation of transcription [10,35]. In the context of leukemia, this global hyperactivation of Mediator function results in cancer cell death [35]. Intriguingly, in pluripotent stem cells, we found that CDK8/19-inhibition and global hyperactivation of Mediator did not result in transcriptional dysfunction, instead, a coherent shift occurred in the transcriptional program, transitioning from one cell identity to another [10]. This raises the possibility of pharmacologically-modulating cell identity of non-cancer cells in a non-deleterious manner.

As mentioned above, currently it is not possible to fully understand the mechanism of CDK8/19 during the transcription cycle. Nevertheless, it is useful to consider the temporal order of events when interpreting the literature, and we summarize the role of CDK8/19 below in this order. CDK8/19 can sterically hinder the initial approach of Pol II to the Mediator complex based on several data and crystal structures [18,19,36], and indeed, this was how CDK8 was first identified in yeast [37]. Thus, CDK8/19 must transiently vacate its initial position to permit Mediator-Pol II interaction [8,9,18,19,38]. In yeast, this process involves CDK11-catalyzed phosphorylation of Mediator subunits MED4 and MED27 [39]. Also, we and others have found that CDK8/19 can phosphorylate itself and several other Mediator subunits [10,30,[36]^37^], raising the possibility that the kinase activity of CDK8/19 may somehow influence its structural role hindering the approach of Pol II to the Mediator complex. In reciprocal, extensive Mediator structural rearrangements re-position CDK8/19 with respect to RNA Pol II [18,19,23].

Later in the transcription cycle, following recruitment of RNA Pol II to Mediator, the pre-initiation complex is completed, and CDK8/19-Mediator undergoes further structural re-arrangements to participate in the release of RNA Pol II into productive elongation [8,9,18,19]. CDK8/19 has been reported to phosphorylate RNA Pol II directly *in vitro* [36,37], and to affect Pol II-CTD phosphorylation *in vivo* [[40], [41], [42], [43], [44], [45]]. Also, CDK8/19 is reported to phosphorylate and orchestrate the function of positive and negative elongations factors including NELF, DSIF, TFIIH and the super-elongation complex [8,9]. However, we suggest that these reported roles of CDK8/19-mediated phosphorylation in elongation may be to some extent redundant, gene-specific, and/or compensated by other kinases such as CDK7 and CDK9. In this regard, we and others have used potent CDK8/19 small molecule inhibitors where transcription is modulated but can still proceed and cells are viable [10,30,35,[43], [44], [45], [46]]. Here, it is notable that while the other transcriptional CDKs, namely CDK7 and CDK9, play general roles positively promoting transcriptional elongation, the role of CDK8/19 appears more nuanced. Thus, while chemical inhibition of CDK7 and CDK9 are lethal, abolishing transcription [[47], [48], [49]], CDK8/19 chemical inhibition appears to modulate the transcriptional program, triggering global hyperactivation of Mediator, and increased Mediator-RNA Pol II interaction [10].

In summary, CDK8/19 has an overall effect of repressing RNA Pol II recruitment and, thereby, CDK8/19 kinase inhibition increases recruitment of RNA Pol II by Mediator and stimulates global hyperactivation of transcription of enhancer target genes [10,35].

## 5 Mediator, intrinsically disordered regions, and transcriptional condensates

RNA Pol II is known to participate in discrete membrane-less protein aggregates previously described as transcription factories [[50], [51], [52]]. More recently, studies have elucidated how these factors efficiently co-segregate from the nuclear milieu based on a shared propensity for phase separation, leading to the term transcriptional condensates [24,25,[50], [51], [52], [53]]. Mediator and several other protein elements of the transcriptional machinery contain intrinsically-disordered regions (IDRs) that confer their phase separation [24,25,50,[52], [53], [54]]. Thus, IDRs act as biophysical addresses to localize each protein, and they contribute an essential structural component to stabilize the transcriptional condensate. In particular, approximately half of Mediator's 30-subunits contain conserved IDRs, including CDK8 and CDK19 [54]. This shift in our understanding has been essential to explain the efficient assembly and structural stability of transcriptional condensates, but also their dynamic flexibility to changes in signaling. Specifically, transcriptional condensates possess vulnerability to perturbation via sharp transitions in their phase separation [17], a regulatory feature conferring dual capacity to stably drive high gene expression, or dissolve rapidly, as required. The ability of transcriptional condensates to switch between formation or dissolution may be essential to rapid transcriptional changes [17,22,55]. Pluripotent cells contain a discrete number of particularly large condensates containing Mediator and RNA Pol II that are thought to correspond to SEs [45,47,48]. Post-translational modifications within protein IDRs have been shown to control transitions in phase separation [53]. Thus, given the central role of the CDK8/19-module for Mediator, we speculate that its kinase activity could affect the entry or exit of proteins into the condensates, or even the formation and dissolution of the condensates themselves. In support of this, we recently identified CDK8/19-dependent phosphosites in the Mediator subunit Med1-IDR domain in mouse PSCs [10], a particularly large IDR previously shown to play a crucial role in transcriptional condensates in mouse PSCs [50].

## 6 The naïve and primed PSC states reflect developmental stages

**Fig. 2:**
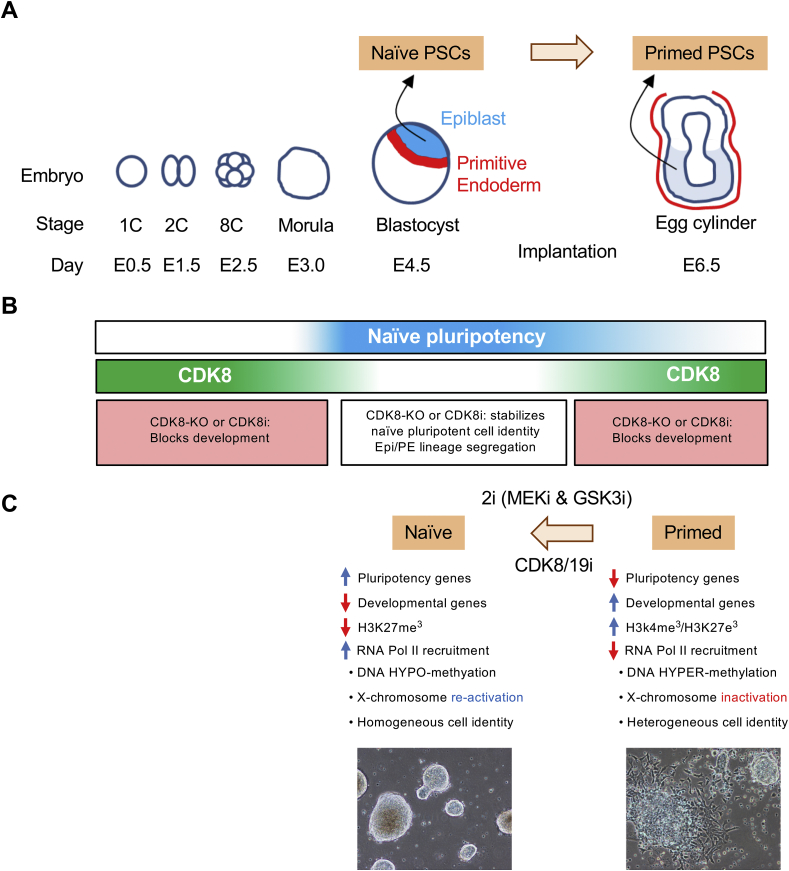
**A summary of CDK8/19 function in early embryo cell identity transitions**. (**A)** Schematic overview of mouse early embryo development. Naïve PSCs can be isolated from the E4.5 epiblast, and they can retain this cell identity homogenously *in vitro*. Primed PSCs can be isolated from the E6.5 epiblast, and they can be maintained in the fluctuating and heterogenous primed state *in vitro*. The 2i inhibitor cocktail can stabilize naïve identity, and promote primed PSCs to transition into the naïve state. **(B)** Similar to small molecule 2i-treatment of PSCs *in vitro*, in the embryo, MEK-signaling is naturally repressed by down-regulation of the FGF receptor. We find that CDK8/19 kinase inhibition, by genetic or pharmacological methods, can phenocopy these effects of 2i: stabilizing naïve identity, and promoting primed cells to transition into the naïve state. Interestingly, we observe that in the embryo, CDK8 activity appears to be naturally repressed, with down-regulation of CDK8 levels and decreased nuclear availability of its essential binding partner Cyclin C. The table below summarizes the developmental phenotypes observed by genetic- or pharmacological-inhibition of CDK8 in the mouse. **(C)** A summary of the molecular and morphological differences reported for naïve and primed pluripotent states.

Pluripotent stem cells (PSCs) transition between cell states *in vitro* which faithfully reflect developmental stages in the early embryo [1,2,6,7] (Fig. 2A–C). Culture conditions to shield mouse PSCs from extra-cellular differentiation stimuli involve chemical inhibition of MEK and GSK3 kinases with a two inhibitor cocktail known as “2i” [6,56]. Mouse PSCs cultured in 2i (referred to as “2i-naïve” cells) phenocopy the stable and homogenous state of undifferentiated naïve pluripotency which exists transiently *in vivo* around the E4.5 mouse pre-implantation embryo epiblast (Fig. 2A–C). In contrast, culture of PSCs with standard serum/LIF media, in the absence of 2i, triggers a shift in cell identity towards post-implantation epiblast, also known as the primed state, analogous to the mouse ~ E5.5–6.5 stage. It is important to clarify that in serum/LIF cells are more properly described as heterogenous, fluctuating between the naïve and primed state [6]. Full primed identity is achieved in FGF/Activin media and this recapitulates the cellular identity of the E6.5 mouse embryo epiblast [1,6,7]. Importantly, PSCs can be maintained during long-term passage in each of these developmental stages. Also, by simply changing the media to alter the signaling inputs to the transcriptional machinery, cells can be directed forward or reverse in developmental identity. In this way, PSCs provide a prototypical model of cellular plasticity, whose transcriptional program can be stabilized, extinguished or re-captured. Notably however, much remains unclear regarding how changes in extrinsic stimuli reset the transcriptional machinery to a new program.

## 7 The transcriptional brain during cellular fate decisions in pluripotency

Here we consider the transcriptional decision-making process. Cell identity is the aggregate outcome of the entire set of active enhancers in a cell at any one moment. During identity transitions, enhancers are activated and decommissioned individually. Thus, the events at each enhancer can be rapid (consistent with the sharp transitions conferred by phase separation), while the aggregate shift in cell identity (all active enhancers) appears relatively slower and incremental. We use as a model the developmental window of naïve-to-primed pluripotency, outlined above. Extensive analyses over the past ~15 years have shown how this path in embryos is followed in close parallel by PSCs *in vitro* [1,2,6,7]. Pluripotency first arises at ~ E3.5–4.5 in mice, and at this initial boundary, cellular identity is very well defined in the naïve state, where cells are effectively deaf to developmental signals and lack any trace of differentiation programs. This is analogous to 2i-naïve PSCs *in vitro* [1,2,6,7,56,57]. Subsequently, cells progress through a gradient of intermediate pluripotency stages, where they gradually become receptive to differentiation stimuli, collectively referred to as being “primed” for development [1,2,6,7]. This period reflects mouse embryo epiblast development from E4.5–5.5. A multitude of studies have captured and defined specific intermediate states with PSCs *in vitro*, referring sequentially, for example, to formative, primed-like, epiblast-like, or rosette states [58] of pluripotency (thoroughly reviewed elsewhere [1,2,6,59]. However, now, and with the benefit of hindsight, we favour the idea that a continuum of pluripotency states exists, marked by incremental changes in gene expression and other detailed molecular characteristics.

**Fig. 3:**
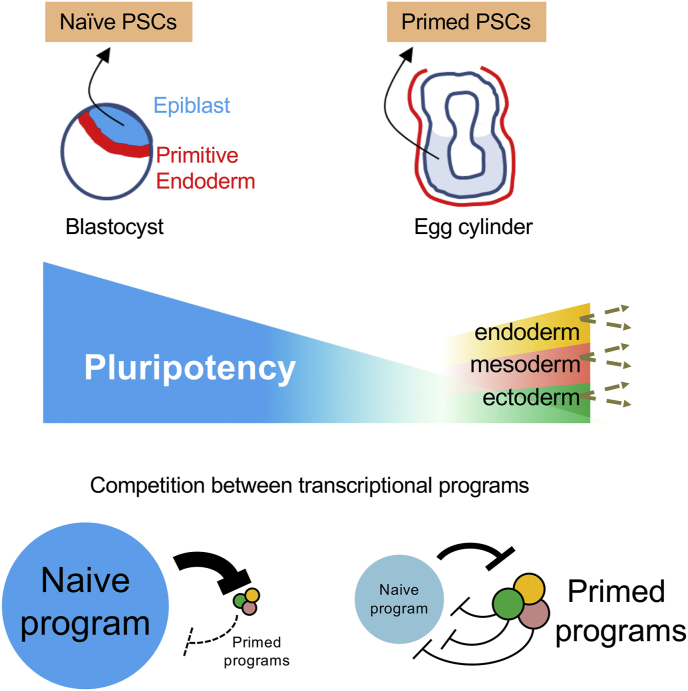
**Emergence and dissolution of transcriptional programs in the naïve to prime transition.** A putative model of dominance and antagonism in transcriptional programs. Binary cell fate choices are common though development and regeneration. In the case of the naïve-primed developmental window of pluripotency, global hyperactivation of enhancers and Mediator-recruitment of RNA Pol II produces a coherent outcome: up-regulation and stabilization of naïve epiblast identity. Here, we suggest a putative mechanism. In primed identity, cells are plastic and heterogenous due to co-existence of lineage-specifying transcriptional programs for forward development into the 3 embryoinc germ layers, however, they are moderately expressed, antagonistic, and weakly established. Powerful naïve-specifiying transcription factors and super-enhancers have begun their decline, but are not yet decommissioned. Upon global hyperactivation of enhancers and Mediator-recruitment of RNA Pol II (by treatment with 2i or CDK8/19i), the naïve-specifiying transcription factors and super-enhancers become dominant, and suppress the nascent germ layer programs.

Ultimately, around E5.5–6.5, mouse embryo epiblast cells exit from pluripotency, and choose between three competing transcriptional programs for lineage-specification, into one of the embryonic germ layers: ectoderm, mesoderm, and endoderm [1,6,7,60] (Fig. 3). This decision-making process remains to be fully understood, however evidence suggests that while naïve cells possess a single homogenous undifferentiated transcriptional program, cells in the primed stages begin to receive simultaneous and competing signals drawing them to activate lineage-specific transcriptional programs of one of the 3 germ layers [[61], [62], [63], [64], [65], [66]] (Fig. 3).

Crucially, lineage-specifying transcriptional programs attempt to reinforce themselves, and repress other competing programs [[67], [68], [69], [70]]. The antagonistic and heterogenous behaviour of transcriptional programs within individual cells at this time is thought to represent a prototypical example of plasticity. Here, cells exist in a super-position, simultaneously expressing the early lineage-specifiers of opposing future fates [[61], [62], [63], [64], [65], [66]]. Thus the cell population is also heterogenous; with individual cells expressing genes that suggest a moderate bias toward one or other fate. Such stochastic decision-making may have evolved to allow cells to sense and respond to diverse environmetal stimuli, ensuring that from one population of cells, three developmental fates emerge. *In vivo*, this is transient, and resolves in a few hours, with each cell choosing a single germ-layer program. *In vitro*, it is possible to capture this decision-making process, as a population of primed PSCs, with heterogenous and fluctuating gene expression programs, literally “priming” themselves to fully-activate ectoderm, mesoderm, or endoderm [1,2,6,7]. It is interesting to consider the events that occur at super-enhancers, and Mediator function, during this competition between transcriptional programs, as these processes may represent common features of many, if not all, transitions in cellular identity. For example, similar co-expression of competing lineage-specifiers has been observed in other fate decisions throughout development and regeneration [71,72]. Thus, the naïve-primed pluripotency window provides a model to elucidate the transcriptional basis of cell plasticity. The molecular lessons learned here are likely to apply to other fate decisions.

## 8 The role of Mediator in pluripotent cell identity

We recently hypothesized that manipulation of the transcriptional machinery during states of plasticity, might toggle cellular identity toward one path or another. We chose PSCs in the naïve-primed pluripotency window as our model of plasticity. As an actionable strategy, we began by targeting the transcriptional CDKs, CDK7, CDK8/19, and CDK9, since these kinases represent direct regulators of the transcriptional machinery, they are amenable to pharmacological inhibition, and indeed, several highly selective small molecule inhibitors exist [10]. We found that selective inhibition of CDK7 and CDK9 produced a general inhibition of transcription, and was ultimately deleterious, consistent with their known general roles in RNA Pol II transcription. In contrast, small molecule kinase inhibition of CDK8/19 (CDK8/19i) produced a striking and characteristic shift in mouse PSC identity, from primed to naïve.

We explored a possible mechanism, following the role of CDK8/19 as a transcriptional CDK, located within the Mediator complex [10]. Also, we compared the induction and stabilization of naïve pluripotency by CDK8/19i versus the well-characterized 2i inhibitor cocktail. 2i is highly-effective in mouse, but not in human PSCs [1,4,73,74], and the effects of 2i on the transcriptional machinery have not been explored. Importantly, we found that 2i-naïve and CDK8/19i-naïve mouse PSCs were highly similar according to their transcriptome and proteome [10]. Collectively, our data suggest that 2i and CDK8/19i rapidly induce a highly overlapping set of phospho-changes focused on the transcriptional machinery (Fig. 1). We also found that Mediator activity was increased in 2i and CDK8/19i, consistent with a repressive role of CDK8/19 in the ability of Mediator to recruit RNA Pol II. Thus 2i and CDK8/19i triggered enhancer hyperactivation, global increase in RNA Pol II recruitment to promoters, and resetting of gene expression. This included the upregulation of enhancer-derived RNAs (eRNAs), and the resetting of endogenous retroviral and repeat element expression. In sum, the ability of 2i and CDK8/19i to induce naïve features appears to originate from their common effect on Mediator and RNA Pol II transcriptional activity. This model is consistent with the concept that transitions in cell identity are driven by early reconfiguration of the active enhancer network, which resets the transcriptional machinery to the new program [14,67,70,[75], [76], [77]]. In summary, targeting Mediator through its kinase module selectively stabilizes an early pluripotent cell identity, repressing differentiation, favoring self-renewal, and up-regulating pre-implantation naïve epiblast gene expression patterns.

As mentioned before, the induction of naïve identity using 2i or CDK8/19i treatments can stimulate Mediator function, which we detect by a global increase in RNA Pol II recruitment, global hyper-activation of existing mouse PSC enhancer loci, and upregulation of enhancer-driven transcription. We propose that this reinforces the pluripotency network underlying naïve PSC identity. In agreement with a recent report [78], we observe that in 2i, mouse naïve-specific enhancer activity is partially resistant to enhancer/Mediator destabilization by BRD4-inhibition. Importantly, this property can also be conferred by expression of CDK8-kinase dead mutant protein. This suggests a simple mechanism where removal of the inhibitory influence of CDK8/19, hyperactivates Mediator function at enhancers, and that this occurs similarly in 2i or via CDK8/19 inhibition. In support of global activation of super-enhancers in the naïve state, a recent study of chromatin looping has revealed that super-enhancers interact with more target promoters, and engage in more long-range interactions, during mouse naïve pluripotency compared to primed pluripotent cells [79], while furthermore, a state of global hyper-transcription has been suggested in PSCs [80,81]. Lastly, we note that the induction of naïve features in human PSCs was recently associated with a significant global increase in the enhancer mark H3K27ac, including at SEs [82]. These data may be consistent with our mechanistic insights, where CDK8/19 inhibition up-regulates enhancer activity, in particular at super-enhancers, thereby driving the stabilization of naïve pluripotency.

## 9 DNA methylation separates CDK8/19i from 2i

**Fig. 4:**
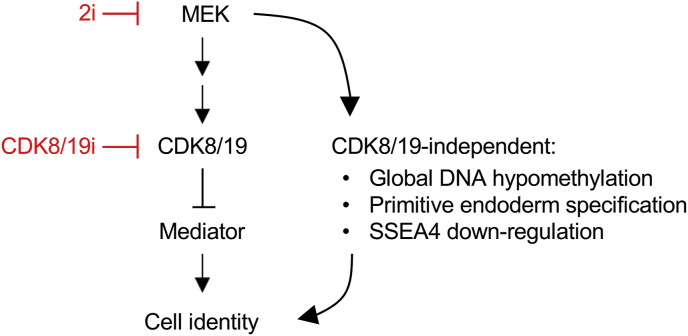
**Unified model of 2i and CDK8/19i naïve pluripotency**. A summary of the signaling hierarchy between MEK, CDK8/19-kinase, and Mediator. A large proportion of the effect of MEK signaling channels through CDK8/19-Mediator to affect the transcriptional program of cell identity. CDK8/19-independent effects of MEK inhibition that we have identified include global DNA hypo-methylation in mouse and human PSCs, specification of the primitive endoderm in the mouse pre-implnatation blastocyst, and, in human PSCs, SSEA4 down-regulation.

As outlined above, CDK8/19i recapitulates a significant proportion of 2i-associated effects on mouse naïve cell identity, however, global DNA hypomethylation was an exception [10] (Fig. 4). The ability of 2i to trigger global DNA hypomethylation is thought to be due to the inhibition of MEK signaling [1,[83], [84], [85], [86], [87]]. It is important to mention that transient DNA hypomethylation is a characteristic of embryonic naïve pluripotency at E4.5 [6]. However, both mouse and human 2i-naïve PSCs differ significantly in the genomic methylation pattern compared to pre-implantation epiblast cells [6,73]. Moreover, it has been reported that 2i-naïve mouse PSCs lose developmental potential after long-term passage [88,89]. This loss of developmental potential has been attributed to the continued inhibition of DNA methylation that resulted in chromosomal instability and loss of imprinting. Moreover, female cells turned out to be more sensitive to the long-term effects of MEK inhibition, consistent with the expression of MEK inhibitory factors from the two active X chromosomes. Transcriptional mechanisms have been proposed to connect MEK-inhibition with DNA demethylation, specifically through upregulation of PRDM14, a transcriptional repressor of the DNA methyl-transferase DNMT3 gene family, and activity of the Tet-family of dioxygenases [83,84,86,87,90].

In contrast to 2i, CDK8/19i recapitulates the same transcriptional changes as 2i, but does not trigger global DNA hypomethylation [10] (Fig. 4). In CDK8/19i-naïve mouse PSCs, DNA methylation remains unchanged. We speculate that MEK inhibition must contribute to DNA demethylation through direct phosphorylation events, such as direct phosphorylation of DNMTs [91]. The DNA hypo-methylation following inhibition of MEK signaling was recently attributed to the down-regulation of UHRF1 protein levels, resulting in a failure to recruit DNMT1 to the replication fork for methylation maintenence [85]. We have confirmed that 2i (which includes MEK-inhibitor) leads to UHRF1 down-regulation, and that this is associated with global DNA hypo-methylation as expected [10]. Interestingly, CDK8/19i treatment did not down-regulate UHRF1 protein levels, thus providing a likely mechanism to explain how CDK81/9i can preserve global DNA methylation [10]. The ability of CDK8/19i to implement naïve pluripotency, without global DNA hypomethylation, may avoid the detrimental side effects of imprint erasure recently reported during extended passage of mouse PSCs under conditions of MEK-inhibition [73,88,89]. Indeed, we have observed that mouse ES cells long-term adapted to CDK8/19i retain full developmental potential upon withdrawal of CDK8/19i, as demonstrated by mouse chimera generation assays, where highly chimeric off-spring developed to adulthood and achieved germline transmission [10]. Since DNA hypo-methylation is part of the naïve stage of pluripotency and is required for normal development [92], the possibility remains that upon withdrawal of CDK8/19i and aggregation of the pre-treated PSCs within a developing blastocyst inner cell mass, a transient wave of DNA hypo-methylation may occur, permitting successful development.

## 10 Application of CDK8/19i for human pluripotency

Species-specific differences exist between human PSCs and our prior understanding of pluripotency based on rodent models [1,5]. In particular, while 2i treatment has a dramatic efficiency in stabilizing naïve features in mouse PSCs, 2i-based media cocktails generally produce extensive cell death during adaption of human and primate PSCs, and are associated with impaired developmental capacity [4,73]. Importantly, CDK8/19i upregulates and stabilizes multiple features of the naïve state in human PSCs, with minimal cell death, shifting the identity of all cells gradually. Cultivation of human PSCs in the presence of a chemical inhibitor of CDK8/19 is sufficient to recapitulate the majority of molecular characteristics associated with a transition from the primed to the naïve state. Other molecular features associated with the more naïve end of this spectrum in human naïve PSCs include SSEA4 down-regulation [73]. However, SSEA4 down-regulation may not be a strict requirement, since there are chemical cocktails that induce a naïve state in human PSCs withouth downregulating SSEA4 [4,93]. Similarly, CDK8/19i also installs naïve features in human PSCs while maintaining SSEA4 (Fig. 4).

## 11 CDK8/19i-naïve human pluripotent cells retain long-term developmental potential

Stabilization of the human naïve pluripotent state *in vitro* has proven to be challenging and remains to be optimized [73,74,94]. In particular the genomic instability and loss of imprinting observed with 2i-induction of induce naïve features are associated with striking loss of developmental potential in human PSCs [73,94,95]. This includes inability, or heavy bias, in forming all 3 embryonic germ layers upon differentiation, for example in embryoid body or teratoma assays, which are normally straight-forward methods for primed human PSCs to express their developmental potency. All this is probably caused, as in mouse, by MEKi-driven DNA demethylation. Indeed, 2i-naïve human PSCs present reduced global methylation, however, the methylation patterns differ significantly from the methylation pattern in pre-implantation epiblast cells [73].

Similar to our observations in mouse PSCs, CDK8/19i does not induce MEK-inhibition or global DNA hypo-methylation in human PSCs [10]. Accordingly, multiple CDK8/19i-naïve human PSCs preserved a normal karyotype after >16 passages [10]. Importantly, upon removal of CDK81/9i chemical inhibition, full developmental potency is maintained [10]. Specifically, following prolonged adaption to CDK8/19i, human PSCs could develop into all three embryonic germ layers in embryoid body and teratomas assays. Moreover, CDK8/19i-pre-treated human PSCs displayed clonal survival and low level chimerism in human-rabbit blastocyst interspecies assays [10]. Altogether, these data suggest that the role of CDK8/19 in pluripotency is conserved in mouse and human, and therefore perhaps across mammalian species.

## 12 The CDK8/19-kinase during early development

Since CDK8/19 kinase inhibition favours up-regulation of naïve pluripotency *in vitro*, we have explored the possible role of CDK8/19 in the embryo, where the naïve state arises naturally in the absence of chemical inhibitors [10] (Fig. 2A). Firstly, we found that CDK8 mRNA and protein expression is ~5×–20× fold higher than CDK19 in mouse and human early embryo development up to day ~ E6.5, suggesting that CDK8, rather than CDK19, is the major player at this stage. Thus we focused on CDK8 function across early mouse embryonic development (Fig. 2B), and its role may be summarized in three periods [10]:

(i) CDK8 is required during 1C to morula development, where its expression is high. In support of this, CDK8-knockout is embryonic lethal before the 4C stage [96], and CDK8/19i blocked development at the 2C stage [10].
(ii) During morula to blastocyst pre-implantation development, CDK8 and cyclin C expression declines. This coincides with the emergence of the E4.5 pre-implantation naïve epiblast and, accordingly, small molecule CDK8/19i does not interfere with naïve epiblast specification [10]. Also, in contrast to MEK inhibition, CDK8/19i does not affect the epiblast/primitive endoderm (EPI/PE) lineage segregation [10] (Fig. 4). In agreement, CDK8-knockout starting in ~E3.5 embryos permitted naïve epiblast specification, and EPI/PE segregation [97]. Specification of the PE is highly sensitive to MEK inhibition [[98], [99], [100]]. The phosphorylation of the transcription factor GATA6 by MEK has been recently shown to be a key event in the determination of the PE [101]. Since CDK8/19i does not affect the kinase activity of MEK [10], it is possible that the presence of an active MEK/GATA6 circuit is sufficient to determine PE formation in the face of CDK8/19 inhibition.
(iii) During the subsequent developmental transition of pre-implantation naïve epiblast to the post-implantation primed state, CDK8 expression becomes increased and its activity is required for the morphogenic events during this transition [10]. Moreover, genetic evidence has very recently emerged of an essential role for CDK8 in post-implantation development around ~ E5.5-E10.5 [97], and we speculate that here upstream signals such as FGF-MEK-ERK may guide CDK8 function (as discussed below in the section on MEK signaling). Therefore, overall, CDK8 function in early embryonic development mirrors its expression pattern (Fig. 2B), with elevated expression and essential roles at the 1–2C stage and during post-implantation development, In contrast, between these periods, a physiological minima in CDK8 expression and function exists around E4.5 that equates to naïve PSCs *in vitro* [10]. In sum, we conclude that the physiological minimum in CDK8 function coincides with the emergence of naïve pluripotent epiblast identity *in vivo*, a feature which can be exploited to stabilize naïve PSC culture by CDK8/19i *in vitro.*

In further support of these data, mouse PSCs are known to include an additional form of transcriptional heterogeneity involving infrequent, but dramatic and transient, re-activation of the gene expression program of the 2C embryo stage [[102], [103], [104]]. It has been shown that 2i treatment represses this 2C fluctuation [102,104], and we have confirmed that CDK8/19i also represses this additional example of transcriptional heterogeneity [10]. We suggest an analogy between the high expression and developmental requirement for CDK8 in the 2C embryo stage, and repression of the 2C transcriptional fluctuation in mouse PSCs by CDK8/19i.

## 13 A signaling hierarchy: CDK8 inhibition downstream of MEK inhibition

Better understanding of the downstream signaling from MEK-ERK, and how it links to the transcriptional machinery, may improve our control over cellular plasticity and aide stabilization of cell identity *ex vivo*. The high degree of overlap in phospho-changes and RNA Pol II regulation induced by 2i and CDK8/19i kinase inhibitors immediately suggests that these chemical inducers of the naïve state might operate within the same pathway [10]. Importantly, analysis of kinase activity has implied a signaling hierarchy (Fig. 1, Fig. 4), where 2i treatment decreases CDK8/19 kinase activity, yet CDK8/19i has no effect on MEK-mediated phosphorylation of ERK. Thus, downstream of MEK-ERK signaling, CDK8/19 activity is down-regulated, explaining how 2i and CDK81/9i treatments functionally overlap in the control of Mediator and the transcriptional machinery [10].

Significant evidence supports the direct input of MEK-ERK signaling into Mediator and the control of the core transcriptional machinery in PSCs. Consistent with this model, it has been recently reported that MEK signalling during exit from the naïve state, directly or indirectly, results in phosphorylation of Mediator subunits and alter eRNA transcription within PSC super-enhancers [32]. All current PSC media cocktails that stabilize the naïve state contain small molecule inhibitors targeting one or more factors in the MEK signalling pathway (FGFRi, RAFi, SRCi, PKCi, p38i, JNKi, MEKi) [1]. It is notable that many components of the MEK pathway have also been shown to regulate CDK8 activity, including KRAS, RAF, SRC, PKC, p38, JNK, MEK, and ERK [[105], [106], [107], [108]]. Thus, CDK8/19-inhibition may be a common feature of media cocktails for stabilizing PSCs in the naïve state. Further studies are required to reveal the precise mechanism by which MEK-ERK signaling regulates CDK8/19 activity in PSCs.

## 14 Model: dominant programs resolve transcriptional decisions in cell identity

A better understanding of the intracellular competition between transcriptional programs may apply widely to human disease. In this regard, global enhancer hyperactivation was recently identified as a common feature across all human cancers tested [109], while addiction to globally up-regulated transcription also appears to be a unifying aspect of cancer [110]. Using the model of PSCs to study these phenomena, we observe that following exposure to 2i or CDK8/19i, the *trans*-activating potential of existing enhancers and Mediator complexes become globally hyper-activated [10]. This raises an important question: why is the naïve pluripotent state favoured by global enhancer/Mediator hyperactivation? A general feature of lineage-specifying transcription factors is that they act to promote their own lineage, but repress alternative fates. This is known to also apply for the transcription factors that specify for the 3 embryonic germ layers as cells exit pluripotency [[61], [62], [63], [64], [65], [66],[111], [112], [113]]. For example, the pluripotency factors OCT4 and SOX2 are known to repress ectodermal- and mesendodermal-lineage enhancers, respectively [65,99,[111], [112], [113]]. Within individual primed cells, these lineage-specifying gene expression programs are in competition with each-other, and they are also moderately expressed and not fully established. Indeed, there is evidence that nascent germ layer programs rely on relatively weak enhancers which are not yet fully established [70,82,114,115], while the naïve enhancers retain a capacity to rapidly return to full strength, retaining their plasticity [10]. Thus, upon global hyperactivation of enhancers by 2i or CDK8/19i, the naïve program becomes dominant, quickly suppressing forward differentiation of the nascent germ layer programs (Fig. 3). In this way, the transcriptional landscape of naïve pluripotency can be up-regulated, establishes dominance, and remains stabilized, by Mediator stimulation, and this can be directly achieved by chemical inhibition of CDK8/19 [10].

## 15 Other applications of Mediator stimulation via CDK8/19i

We note that a similar mechanism of Mediator hyperactivation via CDK8/19 inhibition has been reported in cancer cells [35]. However, intriguingly, this resulted in cell death in acute myeloid leukemia (AML) cells [35], while we find that a similar approach in PSC reinforces naive cell identity [10]. Cancer cells commonly develop novel oncogenic SEs [[110], [116], [117]] that can result in addiction to a defined range of enhancer-driven transcription. Thus cancer cell oncogenic SEs may be sensitive to perturbation, either when hyperactivated, as in the case of CDK8 inhibition [35], or when inhibited, as in the case of BRD4 inhibition [78]. This provides an interesting parallel with MEK inhibition, which is also detrimental to many cancer cells, but is beneficial to the naïve state.

Binary fate decisions are common throughout development and regeneration. We speculate that other cell fate decisions may operate via processes in the transcriptional machinery similar to the naïve-primed pluripotency equilibrium. Thus, we suggest that other examples of CDK8/19i influencing cell identity may exist. Recently, CDK8/19-kinase inhibition was found to produce an anti-inflammatory effect in mouse tissues. This was attributed to the ability of genetic and small molecule CDK8/19-inhibition to promote the production of immuno-suppressive regulatory T cells (T-regs) from their progenitor population of naïve T cells [[118], [119], [120]]. We hypothesize that this process may operate, at least in part, through hyper-activation of enhancers, biasing the outcome of naïve T-cell differentiation toward the production of T-reg cells. It will be interesting to apply CDK8/19i and global hyperactivation of enhancers and Mediator in other examples of cellular plasticity to toggle cell fate, where each system may display a naturally dominant fate.

## 16 Conclusions

Mediator is a central hub (Fig. 1) which we can target pharmacologically using small molecule inhibition of CDK8/19. CDK8/19i removes a repressive influence from Mediator function, effectively triggering hyperactivation of enhancers via the ability of Mediator to more efficiently recruit RNA Pol II [10]. *In vitro*, we identify that CDK8/19i shifts the equilibrium between naïve and primed pluripotent states, favouring naïve features in mouse and human pluripotent cells (Fig. 2). *In vivo*, we observe CDK8 down-regulation in the E4.5 epiblast, coinciding with the natural emergence of the naïve pluripotent state (Fig. 2). Thus CDK8 down-regulation *in vivo* appears to parallel the ability of CDK8/19i to stabilze the naïve state in PSCs *in vitro*.

The effects of CDK8/19i are not dependent on direct MEK-inhibition and this has the advantage of avoiding some deleterious effects of MEK-inhibition such as DNA hypo-methylation and genomic instability in PSCs [10] (Fig. 4). Nevertheless the majority of the other effects of MEK-inhibition can be phenocopied by CDK8/19i^10^. Thus, current evidence suggests that the control of CDK8/19 activity lies downstream of MEK-ERK signaling (Fig. 4). Further studies are required to reveal precisely how MEK-ERK signaling regulates CDK8/19 activity in PSCs. However, we suggest this model to explain how CDK8/19-inhibition can recapitulate many, but not all, molecular events typically observed during the induction of the naïve state downstream of MEK inhibition. Thus chemical inhibition of CDK8/19 offers a new approach that may help to solve remaining challenges in human naïve PSC culture associated with direct MEK-inhibition [10].

Molecular analyses reveal how the RNA Pol II transcriptional machinery is reorganized by CDK8/19i to coordinate cell identity conversion [10]. In the primed state, multiple lineage-specifiying transcription programs are in competition, and we suggest that CDK8/19i resolves this by up-regulating enhancers and forcing one dominant program to suppress the others (Fig. 3). In this way, heterogenous gene expression constituting a plastic cell state, is resolved into a single homogenous expression program and a stable cell identity. This may reveal insights into how the transcriptional machinery resolves other cell fate decisions, and surprisingly, how it can be directly manipulated to produce a coherent outcome, favouring one local cell identity over others. We hypothesize that CDK8/19i may similarly toggle between cell fate outcomes in other systems of cellular plasticity. Indeed, there is some evidence to support this in AML cancer cells, and in T cell differentiation pathways *in vivo*.

The extent to which CDK8/19i mimics 2i suggests a central role of Mediator during the induction of naïve pluripotency, and it provides a mechanism by which naïve pluripotency may arise *in vivo* [10]. Lastly, chemical inhibition of CDK8/19 may help to stabilize other intrinsically unstable cell states, and it will be of interest to transfer these principles to other contexts of cellular plasticity.

## CRediT authorship contribution statement

**Cian J. Lynch:** Methodology, Conceptualization, Writing - original draft, participated in all discussions. **Raquel Bernad:** Methodology, Conceptualization, helped to improve the text and figures. **Isabel Calvo:** Methodology, Conceptualization, helped to improve the text and figures. **Manuel Serrano:** Methodology, Conceptualization, helped to improve the text and figures, Funding acquisition.
